# STAT3 overexpression promotes metastasis in intrahepatic cholangiocarcinoma and correlates negatively with surgical outcome

**DOI:** 10.18632/oncotarget.13846

**Published:** 2016-12-09

**Authors:** Yang Xin-wei, Li Liang, Hou Guo-jun, Yan Xin-zhou, Xu Qin-guo, Chen Lei, Zhang Bao-hua, Shen Feng

**Affiliations:** ^1^ Department of Laparoscopy, Eastern Hepatobiliary Surgery Hospital, Second Military Medical University, Shanghai 200438, China; ^2^ International Co-operation Laboratory on Signal Transduction, Eastern Hepatobiliary Surgery Institute, Second Military Medical University, Shanghai 200438, China; ^3^ Eastern Hepatobiliary Surgery Hospital, Second Military Medical University, Shanghai 200438, China; ^4^ Department of Comprehensive Treatment, Eastern Hepatobiliary Surgery Hospital, Second Military Medical University, Shanghai 200438, China

**Keywords:** intrahepatic cholangiocarcinoma, STAT3, prognosis, proliferation, metastasis

## Abstract

Signal transducer and activator of transcription 3 (STAT3) promotes tumor progression in many types of cancer. In this study, we analyzed the prognostic value of this marker in human intrahepatic cholangiocarcinoma (ICC). Using real-time PCR, western blot and immunohistochemistry assays, we found that STAT3 is overexpressed in ICC patients. STAT3 expression correlated with several clinicopathological features, including tumor size, pathological satellite, vascular invasion, undifferentiated-type histology, lymph node metastasis and TNM stage in two independent cohorts of ICC patients. Patients with high STAT3 levels had a poor prognosis in terms of overall survival (OS) and disease-free survival (DFS). Multivariate survival analysis indicated that STAT3 is an independent prognostic factor for OS and DFS. Furthermore, we observed that STAT3 overexpression promotes the invasion, metastasis and proliferation of ICC cells *in vitro* and *in vivo*, and also promotes STAT3 phosphorylation. These findings suggest that STAT3 expression correlated negatively with surgical outcome and inhibition of STAT3 expression may constitute a novel target for the treatment of ICC patients.

## INTRODUCTION

Intrahepatic cholangiocarcinoma (ICC) arises from malignant transformation of epithelial tissue in the small bile ducts, and is the second most common primary cancer of the liver. The prognosis of ICC is very poor, with the median survival for patients who do not undergo surgery being 6 months, and the 5-year survival rate for patients following complete resection being only 20%–40% [1–4]. In the last decades, the incidence and mortality of ICC has been progressively increasing, while being relatively stable or even slightly decreased for extrahepatic cholangiocarcinoma [2, 3, 5]. The poor prognosis of patients with ICC largely results from the high frequency of recurrence and metastasis after surgical resection, in addition to resistance to systemic chemotherapy [6]. Limited therapeutic options and poor prognosis have triggered a search for molecular markers informative of clinical outcome, which might constitute viable therapeutic targets.

STAT3 is a latent cytoplasmic transcription factor that serves the dual functions of a signal transducer and activator of transcription [7, 8]. STAT3 can be activated by EGFR or IL-6. Once activated by phosphorylation, STAT3 dimerize and translocate to the nucleus, where it activates transcription and in turn modulates cell proliferation, apoptosis and differentiation [7–9]. STAT3 promotes both inflammation and cancer. Constitutive activation of STAT3 has been detected in a variety of primary human epithelial tumors including squamous cell carcinomas (SCCs) of the head and neck, breast, ovary, prostate and lung [10–13]. However, the STAT3 expression level and the significance of which in the prognosis of ICC patients that undergo curative hepatectomy have not been reported. Using a proteomics approach, we identified elevated STAT3 expression in ICC tumor tissues compared with para-tumor specimens. Therefore, it is meaningful to investigate the clinical significance and biological function of STAT3 in the development of ICC.

In addition to investigating the expression pattern of STAT3 and determining its contribution to ICC progression and its clinical prognostic value, we also assessed the functional roles of STAT3 in ICC development and its therapeutic potential. Our results indicate that the expression of STAT3 is significantly correlated with several clinical characteristics and is able to predict the outcome of ICC patients after surgical resection. This cytoplasmic transcription factor may also serve as a therapeutic target for the treatment of ICC.

## RESULTS

### STAT3 is overexpressed in ICC tumor specimens

**Figure 1:**
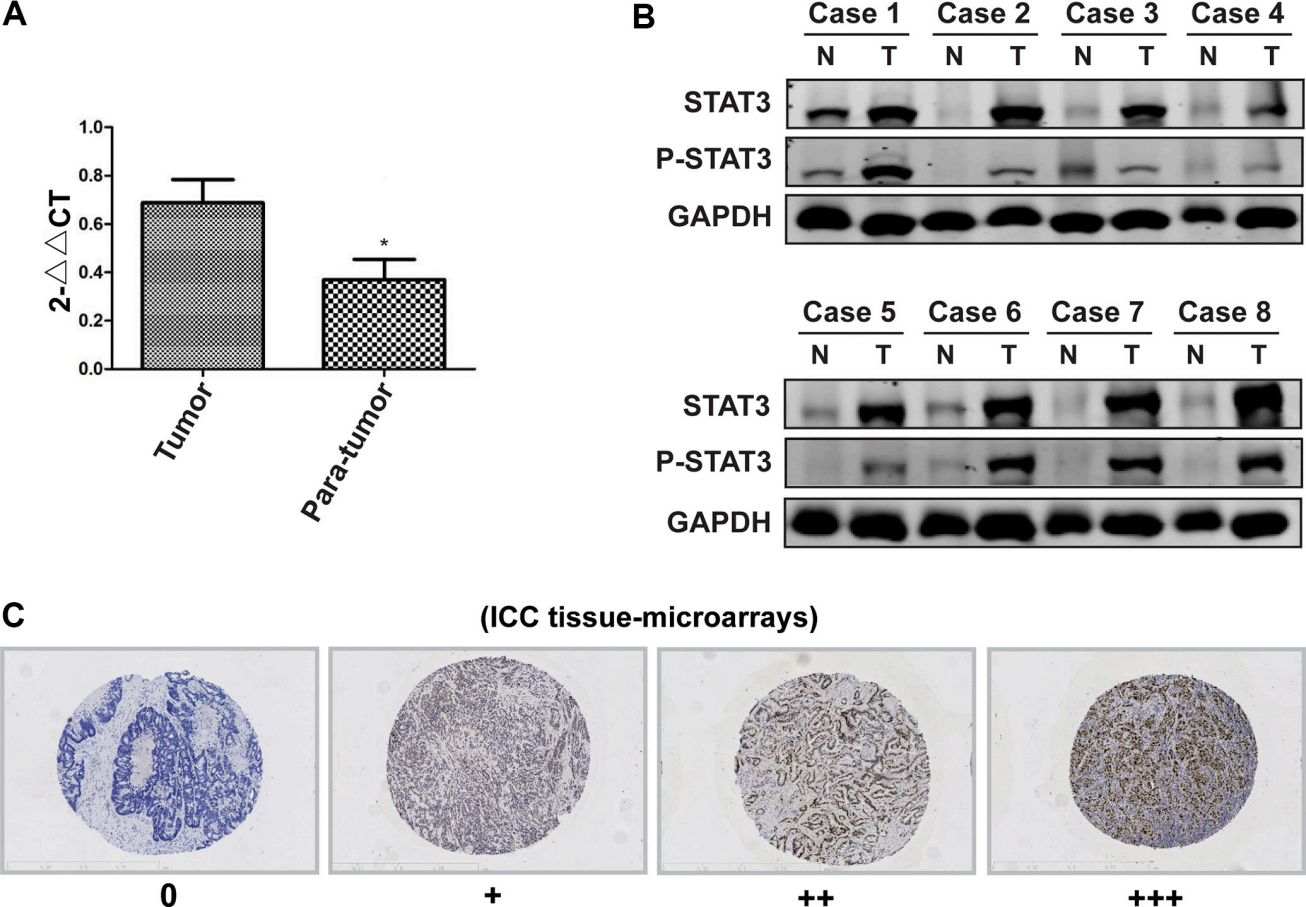
(**A**) The STAT3 mRNA levels in tumor and adjacent liver tissues were detected by real-time PCR. *P < 0.05, compared with tumor group. (**B**) The STAT3 protein levels in tumor and adjacent liver tissues were detected by Western Blot. Representative image is illustrated. (**C**) The TMA assay was performed to detect of the STAT3 expression. The STAT3 was stained with an anti-STAT3 antibody, and the samples were grouped into high and low expression group according to the STAT3 intensity (magnification, ×100).

A real-time RT-PCR assay was performed to analyze STAT3 transcripts in frozen paired samples derived from Cohort A patients with ICC and adjacent liver tissues, with 82% (50 of 61) of tumor tissues having higher expression than the adjacent liver tissues (Figure 1A). The upregulation of STAT3 was confirmed by western blot and immunohistochemical assays (Figure 1B–1C). These results suggested that STAT3 was highly expressed in ICC.

### Association of the STAT3 expression with prognosis

**Figure 2:**
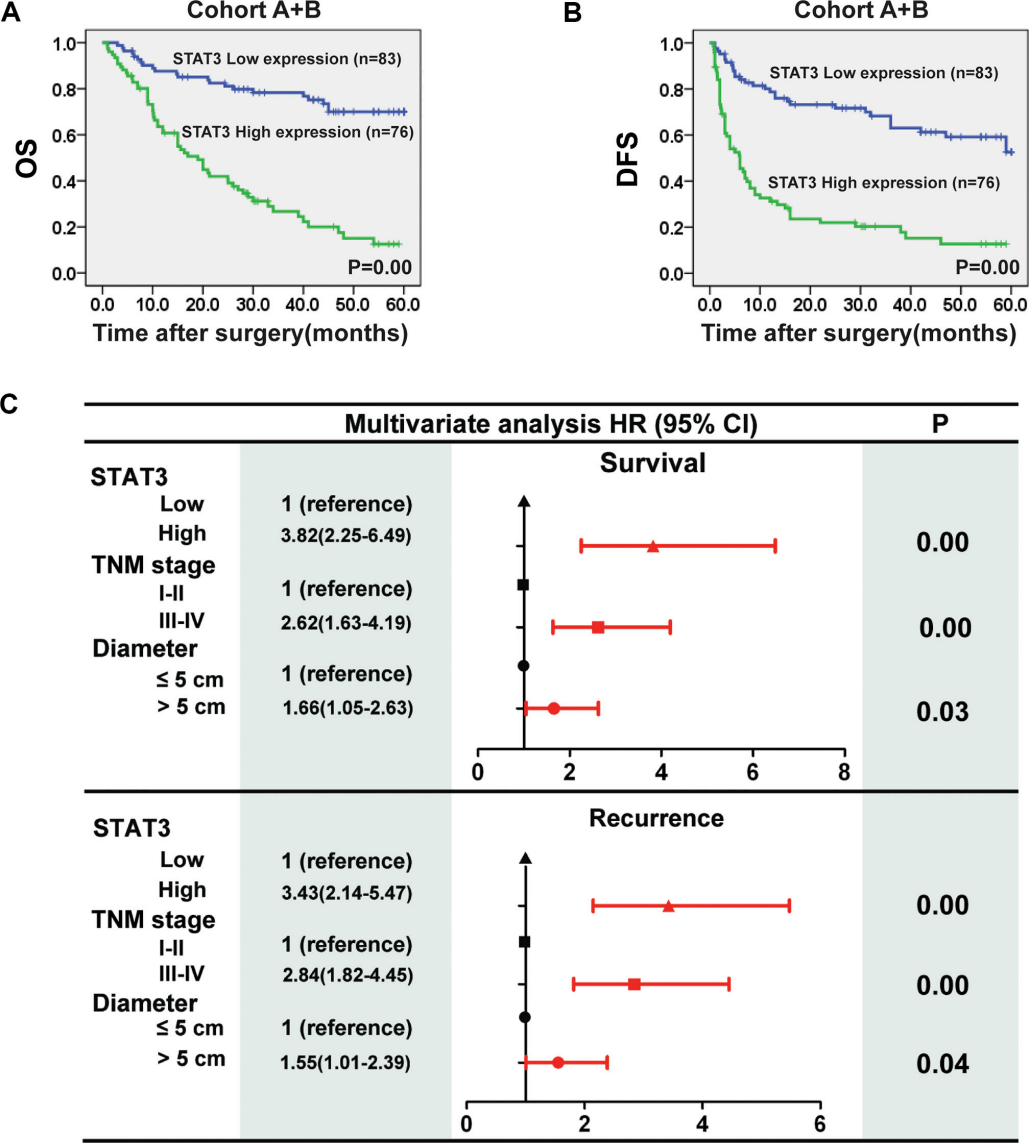
STAT3 expression is an independent prognostic factor for ICC (**A**) Kaplan-Meier analysis was conducted to determine the relationship between overall survival (OS) of patients (Cohort A + B, *n* = 159) with intratumoural STAT3 levels. Subgroups were plotted according to the scores for the STAT3 levels. (**B**) Kaplan-Meier analysis was conducted to determine the relationship between disease-free survival (DFS) of patients with intratumoural STAT3 levels. (**C**) Multivariate analysis of hazard ratios for overall survival and tumor recurrence.

Kaplan-Meier curves comparing the survival of ICC patients with high and low STAT3 expression in two cohorts (*n* = 61 for cohort A, *n* = 98 for cohort B) are shown in Figure 2 and Figure S1. STAT3 expression correlated negatively with overall survival (OS) and disease-free survival (DFS) of ICC patients (Figure 2A– 2B, Figure S1A–S1D). When pooling all the patients from both cohorts together (*n* = 159), univariate analysis revealed that the hepatitis C virus (HCV) infection, tumor size, pathological satellite, lymph node metastasis, TNM stage and STAT3 levels were predictors of OS. Furthermore, the alpha fetoprotein (AFP), tumor size, pathological satellite, microvascular invasion, TNM stage and STAT3 levels were statistically correlated with DFS (Table 1). These significative parameters were further subjected to multivariate Cox proportional-hazards model, which demonstrated that the tumor size, TNM stage and STAT3 expression level were correlated strongly with OS and DFS (Figure 2C). Multivariate survival analysis indicated that STAT3 expression was an independent prognostic factor for OS and DFS (Figure 2C).

**Table 1:** Univariate analysis of clinical variables associated with recurrence and survival

Variables	No	OS		DFS	
		median time to event	P	median time to event	P
Age:					
≤ 50 years	75	39.39 ± 2.75	0.18	31.57 ± 3.08	0.32
> 50 years	84	35.25 ± 2.56		27.86 ± 2.92	
Sex:					
Female	32	40.41 ± 4.47	0.42	37.744 ± 5.006	0.13
Male	127	36.59 ± 2.06		27.973 ± 2.314	
Total bilirubin:					
≤ 17.1μmol/ L	126	36.81 ± 2.10	0.62	28.76 ± 2.37	0.45
> 17.1μmol/L	33	38.69 ± 4.20		33.09 ± 4.73	
ALB:					
≤ 40g/ L	67	35.64 ± 3.09	0.60	33.56 ± 3.43	0.17
> 40g/L	92	38.38 ± 2.34		26.88 ± 2.66	
ALT:					
≤ 40U/ L	111	37.04 ± 2.24	0.99	27.93 ± 2.48	0.27
> 40U/L	48	37.43 ± 3.46		32.83 ± 3.90	
AST:					
≤ 40U/ L	116	35.86 ± 2.17	0.19	27.55 ± 2.41	0.16
> 40U/L	43	40.90 ± 3.68		35.60 ± 4.25	
AFP:					
< 20 μg/L	121	38.41 ± 2.18	0.16	31.88 ± 2.42	0.03
≥ 20 μg/L	38	33.37 ± 3.59		21.68 ± 4.20	
CA19-9:					
≤ 37 U/ml	63	37.63 ± 3.03	0.68	30.40 ± 3.39	0.50
> 37 U/ml	96	36.83 ± 2.39		28.56 ± 2.73	
CEA					
≤ 5 ng/ml	53	36.25 ± 3.37	0.83	28.77 ± 3.64	0.78
> 5 ng/ml	106	37.47 ± 2.27		29.80 ± 2.64	
HBeAg:					
Negative	126	37.39 ± 2.17	0.56	30.56 ± 2.41	0.38
Positive	33	35.94 ± 3.61		24.31 ± 3.93	
HBsAg:					
Negative	73	36.39 ± 2.81	0.64	30.92 ± 3.14	0.47
Positive	86	37.85 ± 2.53		28.45 ± 2.88	
HCV:					
Negative	130	39.01 ± 2.00	0.04	30.02 ± 2.30	0.39
Positive	29	28.96 ± 4.81		28.35 ± 5.54	
Diameter:					
≤ 5 cm	78	42.92 ± 2.45	0.01	34.68 ± 2.94	0.01
> 5 cm	81	31.53 ± 2.68		24.38 ± 2.96	
Tumor Number:					
Single	131	37.83 ± 2.08	0.44	30.85 ± 2.37	0.13
Multiple	28	35.06 ± 4.32		24.57 ± 4.63	
Pathological satellite:					
No	104	40.68 ± 2.27	0.01	33.21 ± 2.61	0.01
Yes	55	30.93 ± 3.16		22.33 ± 3.40	
Encapsulation:					
No	24	40.05 ± 4.90	0.40	35.10 ± 5.55	0.17
Complete	135	36.67 ± 2.03		28.48 ± 2.28	
Microvascular invasion:					
No	99	39.01 ± 2.34	0.20	33.95 ± 2.66	0.01
Yes	60	33.95 ± 3.12		22.45 ± 3.32	
Cirrhosis:					
No	51	31.86 ± 3.40	0.06	28.23 ± 3.82	0.59
Yes	108	39.79 ± 2.21		30.21 ± 2.55	
Differentiation:					
I–II	123	37.37 ± 2.15	0.75	30.78 ± 2.43	0.26
III–IV	36	36.76 ± 3.85		25.88 ± 4.32	
Lymph node metastasis:					
No	118	40.22 ± 2.19	0.01	31.76 ± 2.52	0.05
Yes	41	29.41 ± 3.32		23.42 ± 3.72	
TNM stage:					
I–II	106	44.67 ± 2.12	0.00	38.09 ± 2.53	0.00
III–IV	53	22.18 ± 2.64		11.31 ± 2.10	
STAT3 expression:					
Low	83	48.45 ± 2.21	0.00	42.35 ± 2.67	0.00
High	76	24.26 ± 2.28		15.29 ± 2.40	

### Association of STAT3 expression with clinicopathological features

**Figure 3:**
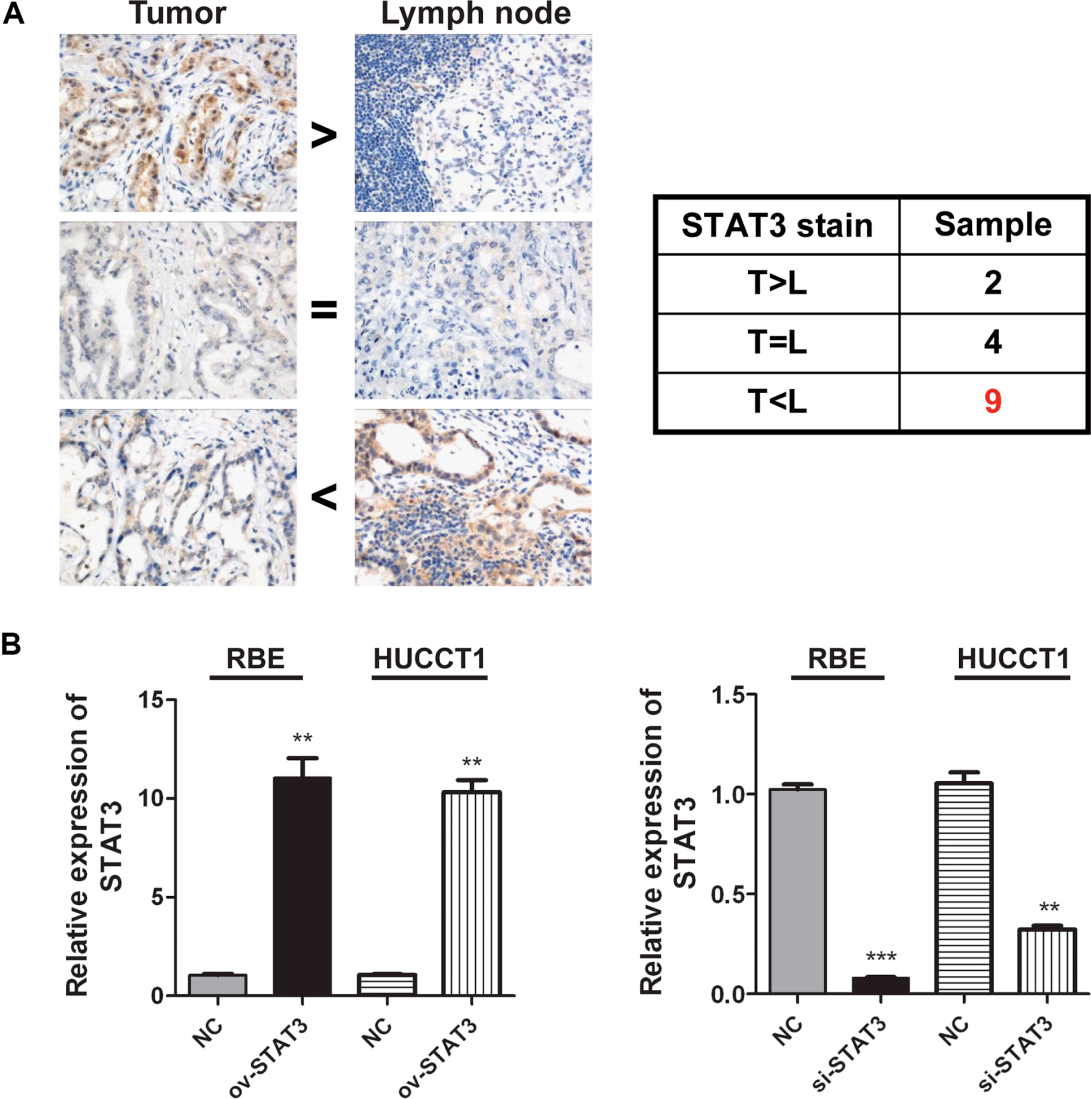
The relationship between the expression of STAT3 and lymph node metastasis (**A**) The expression levels of STAT3 in ICC and relevant lymph nodes were detected by Immunohistochemical test (*n* = 15). Representative image is illustrated. (**B**) The STAT3 expressions were detected by real-time PCR 2 days after over expression or inhibition of it in RBE and HUCCT1 cells, respectively. **P < 0.01, ***P < 0.001, compared with negative control group.

We next examined the relationship between the STAT3 expression in tumor tissues and various clinicopathological characteristics of 159 ICC patients using TMA analysis (Table 2). The expression of STAT3 was significantly correlated with the tumor size, pathological satellite, vascular invasion, undifferentiated-type histology, lymph node metastasis and TNM stage. In the current study, in order to further confirm the relationship between the expression of STAT3 and lymph node metastasis, we examined STAT3 expression in 15 ICC primary tumor and lymph node metastasis tissues (Figure 3A). The expression of STAT3 was higher in metastasis lymph node lesions than ICC primary tumor tissues in most cases (9/15), which suggested that high expression of STAT3 correlated with a greater likelihood of lymph node metastasis.

**Table 2:** Relation between STAT3 expression level and the clinicopathologic characteristics of ICC patients

Variable	STAT3 expression		
	Low (0, 1)*n* = 83	High (2, 3) *n* = 76	*P* value
Age (years)*	50 (29–77)	52 (32–70)	0.30^#^
Sex, M:F	67:16	60:16	0.78
Total bilirubin (umol/L)*	13 (6–438)	13(7–38)	0.18
ALB (g/L)*	43 (27–52)	43(29–52)	0.89
ALT (u/L)*	28(6–132)	34(5–129)	0.81
AST (u/L)*	30(12–113)	33.5(9–111)	0.31
AFP (μg/L), < 20:≥ 20	68:15	53:23	0.07
CA19-9, ≤ 37:> 37 U/ml	36:47	27:49	0.31
CEA, ≤ 5:> 5 ng/ml	29:54	24:52	0.65
HBeAg, positive: negative	15:68	18:58	0.38
HBsAg, positive: negative	46:37	40:36	0.72
HCV, positive: negative	15:68	13:63	0.87
Diameter (cm), ≤ 3: 3–5: > 5	25:25:33	12:20:44	0.04
Tumor number, single: multiple	70:13	61:15	0.50
Pathological satellite, yes: no	23:60	33:43	0.04
Encapsulation, complete: no	14:69	10:66	0.92
Vascular invasion, yes: no	25:58	35:41	0.04
Cirrhosis, yes: no	58:25	50:26	0.58
Differentiation, I:II:III:IV	17:56:8:2	7:43:19:7	0.01
Lymph node metastasis, yes: no	12:71	29:47	0.00
TNM stage, I–II: III–IV	64:19	42:34	0.00

* Median (range). ^#^Mann-Whitney test.

Note: *P* < 0.05 by Chi-square test or Student *t* test

### STAT3 expression promotes the migration of ICC cells

**Figure 4:**
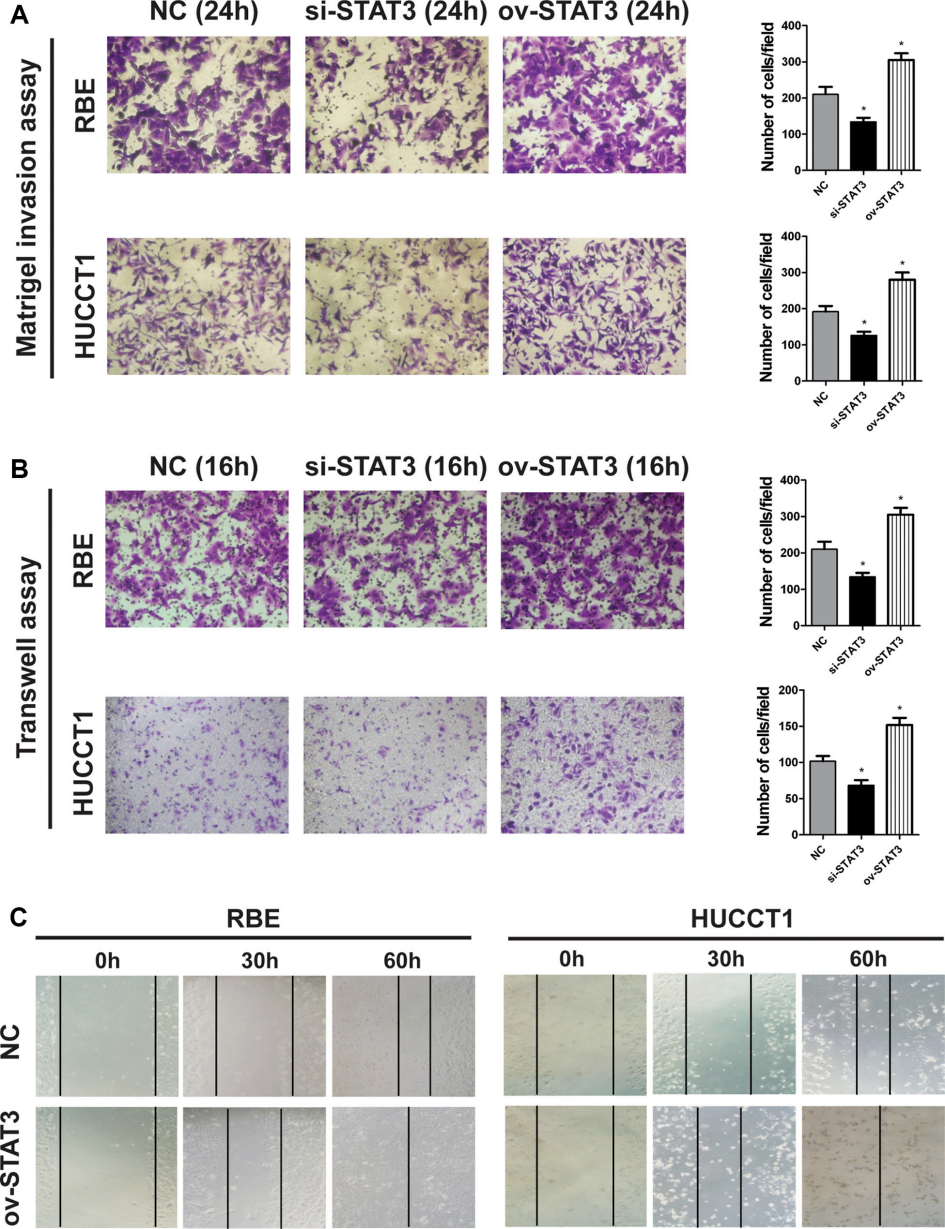
The overexpression of STAT3 enhances the metastatic potential of ICC cells (**A**–**B**) The RBE and HUCCT1 cells were plated into the upper chambers of polycarbonate transwell filter chambers coated with or without matrigel and incubated for 24 and 16 h, respectively. Representative images are illustrated. (magnification, ×200). *P < 0.05, compared with negative control group. (**C**) The wound-healing process of RBE and HUCCT1 cells were estimated by an inverted light microscope after an incubation of 30 h and 60 h.

To further explore the relationship between STAT3 expression and the metastasis capacity of ICC, we constructed STAT3 overexpression plasmids. The ICC cell lines RBE and HUCCT1 were transfected with such plasmids *in vitro* (Figure 3B), and were then used for scratch test and transwell experiments. Figure 4A–4C shows that the overexpression of STAT3 resulted in increased cell invasion and migration of ICC cells compared with control, demonstrating that STAT3 overexpression promotes ICC metastasis. Meanwhile, when we inhibit STAT3 expression with siRNA, the metastasis capacity of ICC cells was blocked (Figure 4).

### STAT3 expression promotes the proliferation of ICC cells and activates the STAT3 phosphorylation

**Figure 5:**
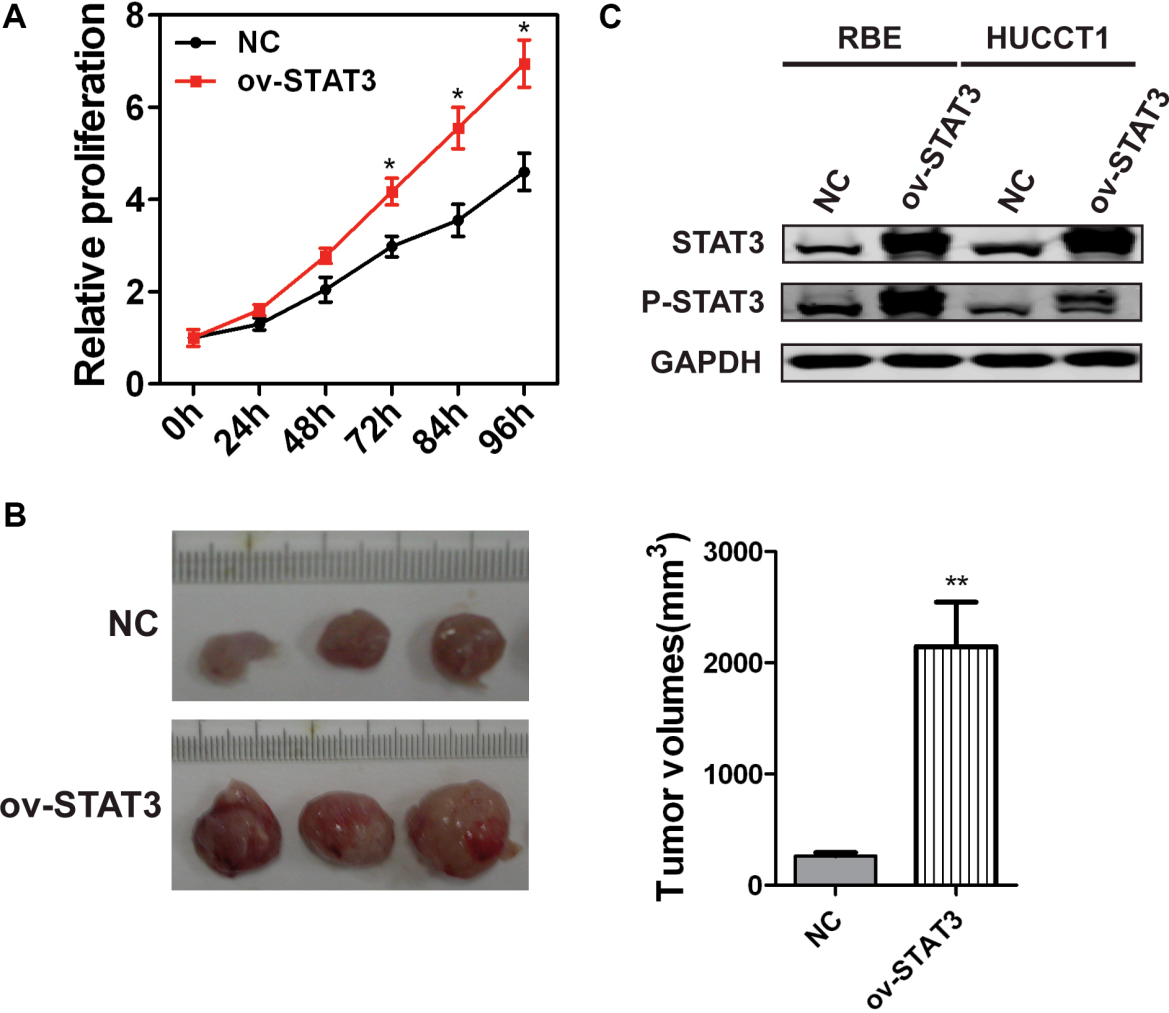
The overexpression of STAT3 enhances the proliferation potential of ICC cells (**A**) Proliferation of STAT3 overexpressed RBE cells was evaluated by a CCK8 assay in indicated time points. *P < 0.05, compared with negative control group. (**B**) The volumes of the subcutaneous tumors developed from RBE cells were significantly increased by STAT3 overexpression. *N* = 3 for each group. **P < 0.01, compared with negative control group. (**C**) The phosphorylation of STAT3 was detected 72 h after STAT3 overexpression in RBE and HUCCT1 cells, respectively.

When compared with negative controls, RBE cells overexpressing STAT3 showed increased proliferation ability *in vitro* and *in vivo*, as estimated by CCK8 and subcutaneous tumor-burdened assays, respectively (Figure 5A–5B). To further study the underlying mechanism by which STAT3 overexpression promotes proliferation and metastasis of ICC cells, we measured STAT3 phosphorylation. The results shown in Figure 5C and Figure 1B suggest that STAT3 overexpression accelerated the progression of ICC by activating STAT3 phosphorylation.

## DISCUSSION

STAT3 levels have been reported to correlate with prognosis for many types of cancers, such as acute myeloid leukemia [17], breast cancer [18], and anaplastic astrocytomas [19], among others. Herein, we demonstrated that STAT3 expression was upregulated at both the mRNA and protein levels in ICC samples. We also investigated the association of STAT3 expression with clinicopathological features in two independent cohorts of ICC patients. We found that high STAT3 expression correlated with multi malignant clinicopathological characteristics, including gross tumor size, pathological satellite, vascular invasion, undifferentiated-type histology, lymph node metastasis and TNM stage. On the other hand, Kaplan-Meier analysis showed that ICC patients with lower STAT3 expression had a better outcome. Furthermore, multivariate analysis revealed STAT3 expression to be an independent prognostic indicator of survival after surgical resection. Therefore, our results suggest that STAT3 expression should be used as a prognostic biomarker for disease outcome in ICC.

Lymph node metastasis has been reported as a prognostic factor of poor prognosis in ICC [12, 14, 15, 17, 20–24]. In our previous study [24], multivariate analysis showed that lymph node metastasis correlated with both the OS and DFS of ICC patients. The median survival time of ICC patients without lymph node metastasis was longer than that of those with lymph metastasis (23 months *vs* 8 months). The presence of lymph node metastasis correlated with other poor prognosis factors, such as gross type of tumor, poorly or undifferentiated tumor, vascular invasion, and perineural invasion. In the current study, we found that the expression of STAT3 was higher in lymph node lesions than that in ICC primary tumor tissues in most cases, which suggested that high STAT3 expression correlated with a greater likelihood of lymph node metastasis. *In vitro*, the overexpression of STAT3 increased cell invasion and migration of ICC cells compared with controls. Thus, high STAT3 expression was an indication of more lymph node metastasis and increased tumor burden *in vivo* and *in vitro*.

STAT3 acts as a latent transcription factor that primarily mediates signaling from cytokine and growth factor receptors [7, 8, 17–19], which results in the phosphorylation of STAT3 and triggers a transcriptional response favoring survival, proliferation and angiogenesis. Accordingly, aberrant and persistent STAT3 phosphorylation is frequently observed in human epithelial origin cancers and is often associated with poor outcome. Our results suggested that high STAT3 expression in ICC patients may facilitate tumor progression and worsen surgical outcomes by promoting its own phosphorylation.

Given the lack of effective adjuvant treatments for ICC, radical surgery is still commonly performed. However, prognosis remains dismal for ICC patients owning to the high recurrence rate after radical resection. In the present study, we found that high expression of STAT3 correlated with tumor progression and poor prognosis in ICC, suggesting that STAT3 expression promotes proliferation and metastasis in ICC. We propose that STAT3 markers could be used in the clinic to classify ICC patients. Furthermore, inhibition of STAT3 expression may offer a novel promising target for the treatment of ICC.

## MATERIALS AND METHODS

### Patients

Two independent cohorts of ICC patients undergoing curative surgical resection were enrolled in this study. Cohort A, with 61 adult patients, was recruited from January 1, 2006 to December 30, 2008 by the Department of Comprehensive Treatment at the Eastern Hepatobiliary Surgery Hospital of the Second Military Medical University. The follow-up period for all patients continued through January 2010. Cohort B, with 98 adult patients, was recruited from January 1, 2009 to December 30, 2012 by the Department of Laparoscope at the Eastern Hepatobiliary Surgery Hospital of the Second Military Medical University. The follow-up period for all patients extended through January 2014. Permission from the Second Military Medical University's Institutional Review Board was obtained prior to data review. Written informed consents were obtained from all the patients for surgical treatment and pathological examinations according to the institutional guidelines. The histological grade of the tumor was defined according to the Edmondson grading system. Tumor staging was performed according to the 7th edition of the tumor-node-metastasis (TNM) classification of the International Union Against Cancer [14].

The specific materials included in this study were selected to include patients who underwent curative surgery without chemotherapy or radiotherapy at a time when these adjunctive therapies were not the standard of treatment. Paraffin embedded tumor tissues were available for the patients included in this study. The follow-up period was defined as the interval from the date of surgery to the date of death or the last follow-up. Patients who died from other causes were treated as censored cases. Overall survival (OS) was defined as the interval between the dates of surgery and death. Disease-free survival (DFS) was defined as the interval between the dates of surgery and recurrence; if recurrence was not diagnosed, patients were censored at the date of death or the last follow-up.

### Tissue microarray and immunohistochemistry analysis

The 5 μm-thick tissue sections of surgically resected intrahepatic cholangiocarcinoma were deparaffinized in xylene and hydrated with graded ethanol and distilled water. Immunohistochemical staining was performed employing the Dako Cytomation Envision-System-HRP (DAB) (Dako Cytomation, Carpinteria, CA) as described previously [15]. The following steps were performed at room temperature unless otherwise specified. Briefly, after inhibition of endogenous peroxidase activity with blocking solution containing 0.03% H_2_O_2_ supplied by the kit for 5 minutes, the sections were incubated in blocking buffer (5% normal goat serum in phosphate- buffered saline containing 0.05% Tween-20) for 30 minutes, and incubated over-night with mouse monoclonal STAT3 antibody (sc-8019) diluted 1:200 with blocking buffer at 4°C overnight. For negative control, the primary antibody was replaced with normal mouse immunoglobulin. Finally, the visualisation signal was developed with diaminobenzidine, and the slides were counterstained with haematoxylin. Stained sections were evaluated in a blinded manner without prior knowledge of the clinical information using the score system as described previously [16]: 0, negative staining (0–10% positive); +, weak signal (10–20% positive); ++, intermediate signal (20–50% positive); +++, strong signal (> 50% positive). Cases with discrepancies in the scores were discussed together with other pathologists until a consensus was reached.

### Overexpression and inhibition of STAT3

The full-length STAT3 plasmid (pcDNA3.1-STAT3) was constructed by inserting a PCR-amplified full-length STAT3 fragment into the NheI/HIND3 sites of pcDNATM3.1 vector (Invitrogen), using the primers, forward, 5′- CTAGCTAGCATGGCCCAATGG AATCAGCTACAGC-3′ and reverse, 5′- CCCAAGCTT CATGGGGGAGGTAGCGCACTCCGAG-3′. ICC cells were transiently transfected using PEI (Poly plus) according to the manufacturers’ instructions.

The siRNA for STAT3 was purchased from Biomics biotechnologies Co. (Shanghai, China). The sense sequence is: GGGACCUGGUGUGAA UUAU dTdT. The siRNA transfection was performed with INTERFERin reagents (Poly plus) according to the manufacturers’ instructions. Briefly, for each well (6 well, for example), dilute 11 μl (20 μM storage concentration) siRNA duplexes into 200 μl of medium without serum. Mix by pipetting up and down. Then, add 12 μl of INTERFERin into the 200 μl of siRNA duplexes, homogenize by vortex immediately for 10 seconds. Incubate for 10 minutes at room temperature to allow transfection complexes to form between siRNA duplexes and INTERFERin. Then, add 200 μl of transfection mix into the 2 ml cell culture medium to complete a final concentration of 100 nM siRNA. Finally, homogenize by gently swirling the plate.

### Cell culture, invasion and metastasis assays

RBE, HUCCT1 cell lines were purchased from Cell Bank of Type Culture Collection of Chinese Academy of Sciences. Cells were routinely maintained at 37°C in an atmosphere containing 5% CO2 in RPMI 1640 Medium, supplemented with 10% fetal bovine serum (FBS, Gibco). Cell migration and invasion assays were performed using the transwell filter champers (Costar, Corning) and BioCoat Matrigel invasion chambers (BD Biosciences) according to the manufacturers’ instructions. Briefly, ICC cells (1 × 10^5^ cells for Transwell assays and 2 × 10^5^ cells for Matrigel assays) were resuspended in serum-free medium, and added into the top chamber. Medium with 10% FBS was added to the lower chamber. Following 16 h (Transwell assays) or 24 h (Matrigel assays) of incubation, cells on the lower surface of the membrane were stained, photographed, and counted using a microscope for each group. For the wound-healing migration assays, monolayers of cells were plated in 12-well plates. They were wounded by scraping with a 200 μL plastic pipette tip and then rinsed several times with medium to remove any floating cells. The wound-healing process was examined by an inverted light microscope (Olympus, Tokyo, Japan) after an incubation of 30 h and 60 h.

### Statistical analysis

Pearson's χ2 test or Fisher's exact test was used to analyze the relationship between STAT3 expression and the clinicopathologic features. Survival curves were calculated using the Kaplan-Meier method and compared using the log-rank test. The Cox proportional-hazard regression model was used for analysis to explore the effect of the clinicopathological variables and STAT3 expression on survival. Statistical significance was considered at *p* < 0.05. Statistical analyses were performed using SPSS Version 18.0 for Windows (SPSS, Inc., Chicago, IL, USA).

## SUPPLEMENTARY MATERIALS FIGURE



## References

[1] Ben-Menachem T (2007). Risk factors for cholangiocarcinoma. Eur J Gastroenterol Hepatol.

[2] Yamamoto M, Ariizumi S (2011). Surgical outcomes of intrahepatic cholangiocarcinoma. Surg Today.

[3] Farges O, Fuks D (2010). Clinical presentation and management of intrahepatic cholangiocarcinoma. Gastroenterol Clin Biol.

[4] Cardinale V, Semeraro R, Torrice A, Gatto M, Napoli C, Bragazzi MC, Gentile R, Alvaro D (2010). Intra-hepatic and extra-hepatic cholangiocarcinoma: New insight into epidemiology and risk factors. World J Gastrointest Oncol.

[5] Nathan H, Pawlik TM, Wolfgang CL, Choti MA, Cameron JL, Schulick RD (2007). Trends in survival after surgery for cholangiocarcinoma: a 30-year population-based SEER database analysis. J Gastrointest Surg.

[6] Guglielmi A, Ruzzenente A, Campagnaro T, Pachera S, Valdegamberi A, Nicoli P, Cappellani A, Malfermoni G, Iacono C (2009). Intrahepatic cholangiocarcinoma: prognostic factors after surgical resection. World J Surg.

[7] Jarnicki A, Putoczki T, Ernst M (2010). Stat3: linking inflammation to epithelial cancer- more than a “gut” feeling?. Cell Div.

[8] Isomoto H, Mott JL, Kobayashi S, Werneburg NW, Bronk SF, Haan S, Gores GJ (2007). Sustained IL-6/STAT-3 signaling in cholangiocarcinoma cells due to SOCS-3 epigenetic silencing. Gastroenterology.

[9] Yao X, Wang X, Wang Z, Dai L, Zhang G, Yan Q, Zhou W (2012). Clinicopathological and prognostic significance of epithelial mesenchymal transition-related protein expression inintrahepatic cholangiocarcinoma. Onco Targets Ther.

[10] Bharti AC, Shishodia S, Reuben JM, Weber D, Alexanian R, Raj-Vadhan S, Estrov Z, Talpaz M, Aggarwal BB (2004). Nuclear factor-kappaB and STAT3 are constitutively active in CD138+ cells derived from multiple myeloma patients, and suppression of these transcription factors leads to apoptosis. Blood.

[11] Kanda N, Seno H, Konda Y (2004). STAT3 is constitutively activated and supports cell survival in association with surviving expression in gastric cancer cells. Oncogene.

[12] Haura EB, Zheng Z, Song L, Cantor A, Bepler G (2005). Activated epidermal growth factor receptor-Stat-3 signaling promotes tumor survival in vivo in non-small cell lung cancer. Clin Cancer Res.

[13] Liu B, Ren Z, Shi Y, Guan C, Pan Z, Zong Z (2008). Activation of signal transducers and activators of transcription 3 and overexpression of its target gene CyclinD1 in laryngeal carcinomas. Laryngoscope.

[14] International Union (2009). Against Cancer: TNM Classification of Malignant Tumors.

[15] Li Liang, Tang Jing, Zhang Baohua (2015). Epigenetic Modification of MiR-429 Promotes Liver Tumor-Initiating Cells Properties by Targeting Rb Binding Protein 4. Gut.

[16] Lanaya H, Natarajan A, Komposch K (2014). EGFR has a tumour-promoting role in liver macrophages during hepatocellular carcinoma formation. Nat Cell Biol.

[17] Benekli M, Xia Z, Donohue KA, Ford LA, Pixley LA, Baer MR, Baumann H, Wetzler M. (2002). Constitutive activity of signal transducer and activator of transcription 3 protein in acute myeloid leukemia blastsis associated with short disease-free survival. Blood.

[18] Sheen-Chen SM, Huang CC, Tang RP, Chou FF, Eng HL (2008). Prognostic value of signal transducers and activators of transcription 3 in breast cancer. Cancer Epidemiol Biomarkers Prev.

[19] Abou-Ghazal M, Yang DS, Qiao W (2008). The incidence, correlation with tumor- infiltrating inflammation, and prognosis of phosphorylated STAT3 expression in human gliomas. Clin Cancer Res.

[20] Ohtsuka M, Ito H, Kimura F, Shimizu H, Togawa A, Yoshidome H, Miyazaki M (2002). Results of surgical treatment for intrahepatic cholangiocarcinoma and clinico- pathological factors influencing survival. Br J Surg.

[21] Uenishi T, Yamazaki O, Yamamoto T, Hirohashi K, Tanaka H, Tanaka S, Hai S, Kubo S (2005). Serosal invasion in TNM staging of mass-forming intrahepatic cholangiocarcinoma. J Hepatobiliary Pancreat Surg.

[22] Tajima Y, Kuroki T, Fukuda K, Tsuneoka N, Furui J, Kanematsu T (2004). An intraductal papillary component is associated with prolonged survival after hepatic resection for intrahepatic cholangiocarcinoma. Br J Surg.

[23] Nakagohri T, Asano T, Kinoshita H, Kenmochi T, Urashima T, Miura F, Ochiai T (2003). Aggressive surgical resection for hilar-invasive and peripheral intrahepatic cholangiocarcinoma. World J Surg.

[24] Zhou HB, Wang H, Li YQ (2011). Hepatitis B virus infection: a favorable prognostic factor for intrahepatic cholangiocarcinoma after resection. World J Gastroenterol.

