# Supplementary material for: STAT3 overexpression promotes metastasis in intrahepatic cholangiocarcinoma and correlates negatively with surgical outcome
Source: Oncotarget. 2016 Dec 9;8(5):7710–21. doi: 10.18632/oncotarget.13846 (PMC5352354; doi:10.18632/oncotarget.13846)
Supplement: Supplementary file 1 [file oncotarget-08-7710-s001.pdf]

STAT3 overexpression promotes metastasis in intrahepatic cholangiocarcinoma and correlates negatively with surgical outcome

Supplementary Materials

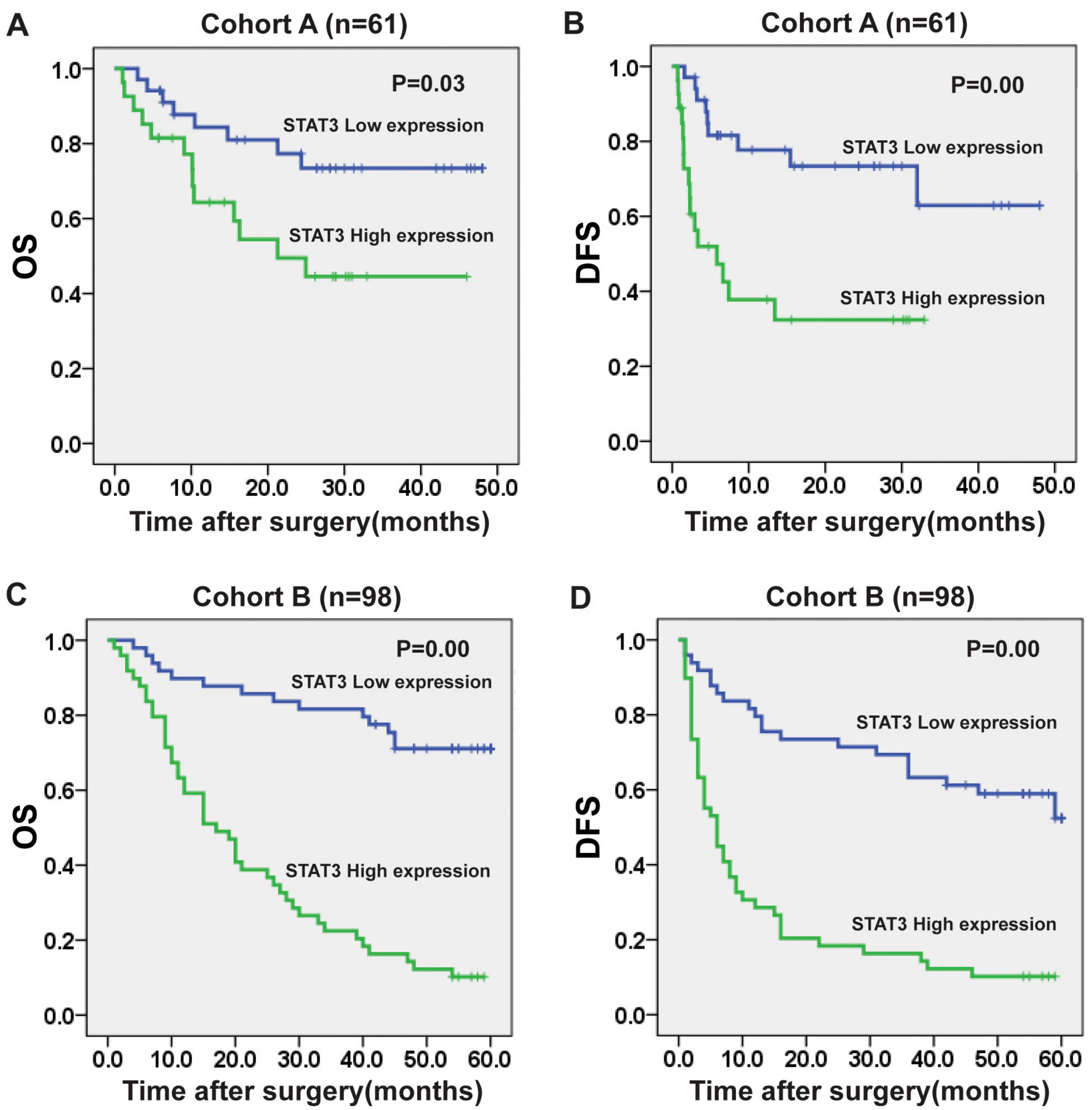

Supplementary Figure S1: The STAT3 expression are correlated with the prognosis of ICC patients in the two independent cohorts. (**A–B**) Kaplan-Meier analysis was conducted to determine the relationship between overall survival (OS) and disease-free survival (DFS) of patients from Cohort A (n = 61). Subgroups were plotted according to the scores for the STAT3 levels. (**C–D**) Kaplan-Meier analysis was conducted to determine the relationship between overall survival (OS) and disease-free survival (DFS) of patients from Cohort B (n = 98). Subgroups were plotted according to the scores for the STAT3 levels.
